# Undetected Toxicity Risk in Pharmacogenetic Testing for Dihydropyrimidine Dehydrogenase

**DOI:** 10.3390/ijms16048884

**Published:** 2015-04-21

**Authors:** Felicia Stefania Falvella, Marta Caporale, Stefania Cheli, Antonia Martinetti, Rosa Berenato, Claudia Maggi, Monica Niger, Francesca Ricchini, Ilaria Bossi, Maria Di Bartolomeo, Elisa Sottotetti, Francesca Futura Bernardi, Filippo de Braud, Emilio Clementi, Filippo Pietrantonio

**Affiliations:** 1 Unit of Clinical Pharmacology, Department of Biomedical and Clinical Sciences, University Hospital “Luigi Sacco”, Università di Milano, Milan 20157, Italy; E-Mails: falvella.stefania@hsacco.it (F.S.F.); cheli.stefania@hsacco.it (S.C.); 2 Medical Oncology Department, Fondazione IRCCS Istituto Nazionale dei Tumori, Via Venezian, Milan 20133, Italy; E-Mails: marta.caporale@istitutotumori.mi.it (M.C.); antonia.martinetti@istitutotumori.mi.it (A.M.); rosa.berenato@istitutotumori.mi.it (R.B.); claudia.maggi@istitutotumori.mi.it (C.M.); monica.niger@istitutotumori.mi.it (M.N.); francesca.ricchini@istitutotumori.mi.it (F.R.); ilaria.bossi@istitutotumori.mi.it (I.B.); maria.dibartolomeo@istitutotumori.mi.it (M.D.B.); elisa.sottotetti@istitutotumori.mi.it (E.S.); filippo.debraud@istitutotumori.mi.it (F.B.); 3 Department of Experimental Medicine, Section of Pharmacology “L. Donatelli”, Faculty of Medicine and Surgery, Second University of Naples, Naples 80138, Italy; E-Mail: bernardi.francesca.futura@gmail.com; 4 Scientific Institute, IRCCS E. Medea, Bosisio Parini, Lecco 23842, Italy; E-Mail: emilio.clementi@unimi.it; 5 Unit of Clinical Pharmacology, Department of Biomedical and Clinical Sciences, Consiglio Nazionale delle Ricerche Institute of Neuroscience, University Hospital “Luigi Sacco”, Università di Milano, Milan 20157, Italy

**Keywords:** single nucleotide polymorphisms, fluoropyrimidines, dihydropyrimidine dehydrogenase, toxicity, pharmacogenetics

## Abstract

Fluoropyrimidines, the mainstay agents for the treatment of colorectal cancer, alone or as a part of combination therapies, cause severe adverse reactions in about 10%–30% of patients. Dihydropyrimidine dehydrogenase (DPD), a key enzyme in the catabolism of 5-fluorouracil, has been intensively investigated in relation to fluoropyrimidine toxicity, and several DPD gene (*DPYD*) polymorphisms are associated with decreased enzyme activity and increased risk of fluoropyrimidine-related toxicity. In patients carrying non-functional *DPYD* variants (c.1905+1G>A, c.1679T>G, c.2846A>T), fluoropyrimidines should be avoided or reduced according to the patients’ homozygous or heterozygous status, respectively. For other common *DPYD* variants (c.496A>G, c.1129-5923C>G, c.1896T>C), conflicting data are reported and their use in clinical practice still needs to be validated. The high frequency of *DPYD* polymorphism and the lack of large prospective trials may explain differences in studies’ results. The epigenetic regulation of DPD expression has been recently investigated to explain the variable activity of the enzyme. *DPYD* promoter methylation and its regulation by microRNAs may affect the toxicity risk of fluoropyrimidines. The studies we reviewed indicate that pharmacogenetic testing is promising to direct personalised dosing of fluoropyrimidines, although further investigations are needed to establish the role of DPD in severe toxicity in patients treated for colorectal cancer.

## 1. Introduction

### Fluoropyrimidines and Their Metabolism

Fluoropyrimidine-based therapy is used extensively in oncology for the treatment of many tumour types including gastrointestinal, breast and the aerodigestive tract cancers. 5-fluorouracil (5-FU) and its oral pre-prodrug capecitabine are the most commonly used chemotherapeutic agents either in monotherapy or in combination regimens [1]. As it occurs with other cancer therapeutics, fluoropyrimidines have a narrow therapeutic range, with the ratio of the effective to toxic dose being small [2]. Severe adverse effects (AE) such as myelosuppression, diarrhoea, mucositis and hand-foot syndrome are still often observed, with grade 3–4 toxicities occurring in 10% to 30% of patients, depending on the regimen used. Severe toxicity can lead clinicians to delay, reduce or interrupt treatment, with consequent negative impacts on patient outcomes. In colorectal cancer (CRC), fluoropyrimidines are often given as part of a regimen that includes other cytotoxic drugs such as oxaliplatin and irinotecan—with or without monoclonal antibodies. This approach while improving the overall therapeutic efficacy is often accompanied by additional toxic effects. In this scenario, the use of pharmacogenetic testing to predict specific toxicity for each drug used could be important to define the overall toxicity risk.

The mechanism of action of the fluoropyrimidines comprises the inhibition of thymidylate synthase and the metabolic impairment of DNA and RNA by incorporation of drug metabolites; the mechanism of action is influenced by the different modes of administration of 5-FU (bolus *vs.* continuous infusion) [3,4,5]. The initial and rate-limiting enzyme in pyrimidine catabolism is dihydropyrimidine dehydrogenase (DPD), which converts 5-FU to dihydrofluorouracil (DHFU). Up to 80% of administered 5-FU is catabolised in the liver, where DPD is abundantly expressed [6]. DPD is encoded by the dihydropyrimidine dehydrogenase gene (*DPYD*) mapping on chromosome 1p21.3 and consists of 23 exons [7]. Most of the adverse reactions to fluoropyrimidines are likely to be the result of inter-individual genetic variation; hence the intense investigation of the role of DPD in them. DPD activity is in fact highly variable in the population and several *DPYD* polymorphisms have been associated with decreased enzyme activity and increased risk of 5-FU severe toxicity. Low or deficient DPD activity was found in at least 3%–5% of individuals [8] representing an autosomal codominantly inherited trait [9]. Unfortunately, results from different studies are often contradictory and the correlation between *DPYD* polymorphisms and 5-FU toxicity is not yet clearly defined.

## 2. Genetic Determinants of Fluoropyrimidine Toxicity

### 2.1. Non-Functional DPYD Variants

To improve the patient’s quality of life from treatment-related toxicity “Clinical Pharmacogenetics Implementation Consortium Guidelines” were drafted [10]; the purpose of these guidelines is to provide dosing recommendations for fluopropyrimidines based on germline variations (DPYD*2A/c.1905+1G>A, *13/c.1679T>G, rs67376798/c.2846A>T) of the *DPYD* gene. Individuals homozygous or compound hererozygous for DPYD*2A, *13, rs67376798 can exeprience severe or even fatal toxicity. Homozygotes for nonfunctional DPYD*2A, *13 or rs67376798 variants have very low enzyme activity or are completely DPD deficient [11,12,13] and, therefore, require the selection of an alternative drug. In the case of patients heterozygous for the nonfunctional *DPYD* variant alleles—with partial DPD deficiency [14,15]—the recommended starting dose is at 50% (or less) of the one recommended in wild type patients. The subsequent dosing should be titrated by dose increases or decreases according to drug tolerability and toxicity. The Minor Allele Frequency (MAF) of DPYD*2A, *13 or rs67376798 variants in all population is estimated to be <0.01 (1000 genome project phase 1 [16]). The positive and negative predictive values of DPYD*2A, *13 and rs67376798 genotyping to predict severe toxicity are ~62% and ~95%, respectively; the sensitivity is 31% [10]. Lee and colleagues recently found low sensitivity (5%) and negative predictive values (68%) of DPYD*2A, *13 and rs67376798 genetic testing for grade ≥3 5FU-AE, possibly because of the combination of chemotherapy regimen in their population that results in additive effects on AE [17].

In the largest study to date, Lee *et al.* [17] found a statistically significant association between DPYD*2A and c.2846A>T variants and >3 grade 5FU toxicity in 2886 patients treated with adjuvant FOLFOX or FOLFIRI with or without cetuximab. In detail, this association remained significant after adjusting for age, sex, grade, tumor stage, performance status, tumour location, KRAS status, microsatellite instability number of cycles, dose modification and treatment, confirming the importance of *2A e c.2846A>T in predicting toxicity when FOLFOX or FOLFIRI regimens were administered.

In a recent meta-analysis of 16 published studies, Rosmarin *et al*. [18] found association only between DPYD*2A and c.2846A>T variants and capecitabine monochemotherapy toxicity. DPYD*2A and c.2846A>T also showed a trend to greater toxicity with 5-FU monotherapy, mainly because of diarrhea with infusional administration and neutropenia for bolus administration of the drug.

### 2.2. DPYD Undetected toxicity

The goal of pharmacogenetics testing is to minimise severe toxicity and obtain the maximum therapeutic efficacy. Genotyping of non-functional *DPYD* variants before treatment decision may help clinicians to drive dosing personalisation of fluoropyrimidines in individuals heterozygous for DPYD*2A, *13 and c.2846A>T and to select an alternative drug in homozygotes. Once the initial fluoropyrimidine dose administration has been reduced according to *DPYD* genotypes, the dosing needs to be further titrated based on tolerance or toxicity.

This is a critical issue; whereas the genotyping of non-functional germline variations is strongly recommended to avoid toxicity, the absence of any of the variants does not equate with absence of DPD deficiency. The *DPYD* gene is highly polymorphic and therefore additional *DPYD* variants may contribute to DPD deficiency. Patients without DPYD*2A, *13 and c.2846A>T alleles can still develop severe toxicity during fluoropyrimidine treatment. Due to the polyallelic mechanisms of DPD deficiency, a comprehensive predictive screening for fluoropyrimidines toxicity is complex and available information is still not complete. In fact, the most promising *DPYD* variants are not validated for clinical use, because of the retrospective nature of most studies, which lack of pre-specified biomarker subgroup analyses. In addition, the limited sample size of most studies poses the challenge of validation to be carried out in larger data-sets of patients receiving homogeneous treatments. This is a critical issue with no easy solution given the heterogeneity of treatment protocols according to the drugs used and since the treatment intent (curative *vs.* palliative) may influence outcomes. Toxicity may also be underestimated because of the intrinsic nature of the trials themselves as they tend to include patients with optimal perfomance status, low median age and liver laboratory tests within the normal ranges, thus minimising the risk of severe toxicity.

Impact of sex and ethnicity on 5-FU related toxicity has been often investigated. Females experienced more severe toxicity than males during 5-FU treatment [19]. Lee and colleagues recently observed a greater effect of DPYD*2A in males compared with females (unadjusted OR = 20.96 *vs.* 9.74), in Caucasian patients, however the sex-gene interactions were not statistically significant [17]. Further studies in larger female populations with equal representation of DPYD*2A will be proposed to investigate the observed difference between male and female DPYD*2A carriers. Regarding ethnicity, DPYD*2A and c.2846A>T variants are more frequent in Asian or European than in African populations, while the *DPYD* rs115232898 (Y186C) variant has been described only in African Americans patients with fluoropyrimidine related toxicity [20].

### 2.3. Common DPYD Polymorphisms

Based on the issue of *DPYD* undetected heritability, additional variants have been evaluated for DPD activity and associated fluoropyrimidine toxicity. Among them, *DPYD* c.496A>G, c.1129-5923C>G and c.1896T>C may have a role, but their use in clinical applications still needs to be validated.

The allelic variant c.496A>G is located in exon 6 of *DPYD* and results in a methionine-valine (M166V) transition. This variant is quite frequent with an overall allele frequency of 7% and 12% in the European population (1000 Genomes Project Phase 1). The c.496A>G variant has been classified either as a variant which is related to DPD deficiency [21] or as a variant accompanied by either normal DPD activity, in peripheral blood cells [9], or higher enzyme activity, in *in vitro* studies [22]. The allelic variant c.496A>G was shown to be strongly associated with grade 3–4 toxicity in patients affected by gastroesophageal and breast cancer, but not for colorectal cancer when treated with a 5-FU-based therapy [23]. Loganayagam reported no association with c.496A>G and fluoropyrimidine toxicity in cancer patients treated with different chemotherapy regimens [24]. Three additional studies failed to confirm a link between this variant and toxicity associated to fluoropyrimidines [25,26,27]. This conflicting evidence may depend on the retrospective nature of the studies, heterogeneity of patients and tumors, as well as chemotherapy combinations. Furthermore an analysis in pooled fluoropyrimidines-treated population demonstrated that the c.496A>G variant protected against overall haematological toxicity and neutropaenia in women [28].

Recently, the deep intronic variant c.1129-5923C>G was found to be significantly associated to severe 5-FU toxicity [29]. Its MAF in all populations is estimated to be <0.01 but increasing up to 2% in Europeans (1000 Genomes Project Phase 1). This variant affects DPD pre-mRNA splicing by creating a cryptic splice donor site leading to the insertion of a 44 bp fragment in the mature DPD mRNA, a new reading frame, and a premature stop codon in exon 11 [29]. The c.1129-5923C>G variant is likely to be in *cis* with c.1236G>A (E412E). Conflicting data exist about an association between c.1236G>A, and an increased risk of severe 5-FU-associated toxicity [25,28]. However, Amstutz and colleagues reported that a haplotype containing three intronic polymorphisms (c.483+18G>A, c.959-51T>G, c.680+139G>A) and the c.1236G>A mutation was associated with severe 5-FU toxicity [26]. Since carriers of the c.1129-5923C>G and c.1236G>A mutation also possessed the three intronic mutations c.483+18G>A, c.959-51T>G and c.680+139G>A, van Kuilenburg and colleagues [29] supposed that the increased susceptibility for 5-FU toxicity of carriers of this haplotype was most likely due to the presence of the causal variant c.1129-5923C>G in accordance with the observation that the c.1129-5923C>G variation was significantly enriched in patients suffering from severe 5-FU toxicity. A significant association with the c.1129-5923C>G variation was also reported by Froehlich and colleagues in a study on 500 patients receiving fluoropyrimidines-based chemotherapy [30].

Finally, a previous study showed that carriers of the *DPYD* 1896T>C variant have increased 5-FU serum concentrations and a higher risk of treatment-related neutropaenia [31].

## 3. Importance of Pharmacogenetic Testing in Patients Receiving Chemotherapy Combinations

Diarrhoea and neutropaenia are the main toxic effects of irinotecan leading to dose-limitations and are also increased when the drug is associated with fluoropyrimidines. Since increased toxicity of chemotherapy combinations remains an unquestionable issue, patient management with adequate supportive measures is a priority for clinicians. The rational use of pharmacogenetics and the personalisation of dosing may allow safe administration of intensive regimens containing both irinotecan and fluoropyrimidines with maximisation of their therapeutic index [32]. It is well known that the UGT1A1*28 allele affects gene expression and leads to decreased glucuronidation of the irinotecan-metabolite SN-38 and increased risk of severe irinotecan-induced AE [33,34]. Thus, concomitant assessment of both *UGT1A1* and *DPYD* may be particularly valuable in this setting; this is particularly true for CRC patients receiving triplet chemotherapy with fluoropyrimidines, oxaliplatin and irinotecan—with or without monoclonal antibodies. Recently, a FOLFOXIRI regimen achieved significantly superior outcomes as compared to standard doublet chemotherapy in terms of response rate, progression-free survival and overall survival [35]. Initially, triplet chemotherapy with FOLFOXIRI plus bevacizumab gained popularity as conversion strategy in potentially resectable CRC liver metastases [36] or in selected, poor-prognosis patient populations, such as those with BRAF-mutated tumours [37]. However, this strategy of upfront treatment intensification was recently established as a palliative treatment option due to improved patient survival [38].

We recently carried out a pharmacogenetic study in 64 advanced CRC patients lacking non-functional *DPYD* variations and receiving triplet chemotherapy with capecitabine, oxaliplatin and irinotecan—with bevacizumab or cetuximab [39]. We simultaneously considered SNPs involved in both irinotecan and fluoropyrimidines metabolism since they are associated with overlapping toxicities, mainly diarrhea. We analyzed only a selected pharmacogenetic panel including the most likely genetic *DPYD* functional variants in association with the UGT1A1*28 allele. We observed a significant association (*p* = 0.021) between *DPYD* c.496 *G* risk allele and grade 3–4 chemotherapy-induced adverse events (AEs), with an OR of 4.93 (95% CI, 1.29–18.87). Using multivariate analysis, we confirmed an independent association of the *DPYD* c.496A>G variant with severe toxicity (*p* = 0.022). Also the UGT1A1*28/*28 homozygous status and the *DPYD* c.1896T>C variant showed a clinically significant association with severe toxicity. We however did not detect any significant association of the *DPYD* c.1129-5923C>G variant with severe toxicity, probably due to the small sample size [39].

Our strategy allowed us to reveal at least part of the genetically-based toxicity which goes undetected in the assessment of only the guideline-recommended non-functional *DPYD* variants. Our analysis provides also a rational approach that opens new windows for investigation. Intensive regimens such as FOLFOXIRI could be reassessed in selected patients populations, such as those with *DPYD* c.496A>G and/or c.1896T>C variants, within the context of a Phase 1b dose-finding study. The validation of results in a prospective large trial is also necessary so that patients at highest risk of toxicity are pre-emptively identified. Genome-wide association studies and next-generation sequencing technologies in large cohorts may strengthen results obtained by candidate gene studies and identify new common or rare risk variants.

## 4. Epigenetic Regulation of DPD Expression

As revealed from the data reported above, variant alleles in the *DPYD* gene are insufficient to explain differences in DPD expression and 5-FU sensitivity. Part of the variable activity of DPD may instead be explained by epigenetic factors; indeed methylation of the promoter, as well as its regulation by microRNAs, (miRNAs) has been shown to occur in *DPYD*.

In an *in vitro* study, Noguchi and colleagues found that DPD activity was in part controlled by aberrant methylation of the *DPYD* promoter region which acted as a repressor of *DPYD* expression [40]. In an *in vivo* study in a small series of clinical samples from DPD-deficient volunteers and DPD-deficient cancer patients, Ezzeldin and colleagues [41] confirmed that methylation of the *DPYD* promoter region is associated with down-regulation of DPD activity. A more recent study that assessed the methylation status of the *DPYD* promoter region by quantitative methylation-specific Polymerase Chain Reaction (PCR) in gastrointestinal cancer patients failed to establish an association of methylation with 5-FU severe toxicity [42]. Additional studies are thus needed to clarify the role of promoter methylation in the regulation of DPD activity.

Recent data in lung tumours have suggested that differences in DPD expression may be arise as a consequence of miRNAs post-transcriptional regulation of the promoter [43]. *In vitro* experiments confirmed that the overexpression of miR-27a and miR-27b in colorectal carcinoma cells reduces DPD expression [44]. In the same report the authors describe that mouse liver DPD enzyme activity was inversely correlated with the expression levels of miR-27a and miR-27b. They also showed that DPD activity was regulated by variant alleles of rs895819, mapping in the coding region of the hsa-mir-27a hairpin. This variant results in a loop region larger than a common hairpin, and so positively influences mature miR-27a. DPD enzymatic activity was lower in volunteers carrying the rs895819 variant allele [44].

We also investigated rs895819 in colorectal cancer patients receiving triplet chemotherapy detecting significant association with 3–4 grade toxicity at least in univariate analysis, not confirmed at the multivariate analysis. Recently, Amstutz *et al.* [45] suggested that within the group of *DPYD* risk variant carriers, the rs895819 genotype may influence fluoropyrimidine toxicity risk. In particular, they found that the correlation of rs895819A>G with fluoropyrimidine toxicity depended on *DPYD* risk variant carrier status so that patients carrying both rs895819G and a *DPYD* risk variant had a strongly increased risk of toxicity. Conversely, in the absence of *DPYD* variants, rs895819G was related to a modest decrease in toxicity risk.

## 5. Conclusions

Fluoropyrimide-based therapies are the standard of care for colorectal cancer patients. In patients with DPD deficiency, 5-FU can cause profound toxicity, such as mucositis, myelosuppression, hand-foot syndrome and diarrhoea. Pharmacogenetics-guided dosing is recommended only for the DPYD*2A, *13 and c.2846A>T variants.

For the other common *DPYD* genetic variants, data are lacking and conflicting. Further research is needed to determine the optimal treatment strategy for patients carrying these *DPYD* risk alleles. Differences between results of studies can be explained by geographic allele frequency variability, by heterogeneity in toxicity assessment and time-points used, and by the heterogeneity in the 5-FU-based chemotherapy regimens. Given the common administration of fluoropyrimidine in combination therapy, investigation of the impact of concomitant cytotoxic drugs on toxicity risk may be enhanced in patients with and without *DPYD* risk variants.

For example, considering that 5-FU is often combined with irinotecan, concomitant assessment of *DPYD* variants and UGT1A1*28 allele could be a strategy for dose personalisation. UGT1A1*28 allele can increase the risk of severe irinotecan-induced neutropaenia contributing to the toxicity of a doublet regimen [32]. It is reported that the combination of DPYD*2A and UGT1A1*28 with concomitant use of fluoropyrimidine and irinotecan can increase toxic effects, resulting also in lethal outcomes in these patients [46]. Fluoropyrimidine dosing based only on pharmacogenetic screening might be misleading because DPD deficiency is not the sole determinant of 5-FU toxicity. Prospective large trials are needed to successfully propose the use of complex pharmacogenetic tests for predicting fluoropyrimidine toxicity and to optimise personalisation of fluoropyrimidine doses in patients with advanced colorectal cancer.
